# The Emergence of Value-Based Leadership Behavior at the Frontline of Management: A Role Theory Perspective and Future Research Agenda

**DOI:** 10.3389/fpsyg.2021.635106

**Published:** 2021-05-25

**Authors:** Sin Mun Chang, Pawan Budhwar, Jonathan Crawshaw

**Affiliations:** ^1^Department of Work and Organisation, Aston Business School, Aston University, Birmingham, United Kingdom; ^2^Department of Management, Leadership and Organisations, Middlesex University Business School, London, United Kingdom

**Keywords:** ethical leadership, servant leadership, authentic leadership, role theory, frontline manager

## Abstract

The importance of value-based leadership such as authentic, ethical, and servant leadership is inconspicuous. However, the benefits of these leadership approaches are often only explained through the behaviors of their followers. As such, limited research has communicated the leader’s motivation for pursuing such leadership behavior, resulting in such discourse to escape theorizing. We draw upon role theory and paid attention to the role of higher-level management (leadership) through the trickle-down model to underline their importance in the organization. We then expand this role theory framework by synthesizing research to explain the emergence of value-based leadership behavior at the frontline of management. In doing so, we aim to provide a stronger explanation of the emergence of value-based leadership in organizations. We conclude this analysis by guiding future research in the form of propositions to investigate the psychological process and organizational factors to empirically examine the proposed role framework.

## Introduction

Leadership is one of the most studied social phenomena that have spanned over more than a century (Rindova and Starbuck, 1997). It is suggested that leadership is a universal activity evident in humankind and animal akin (Bass and Bass, 2008). The attention on value-based leadership (VBL) behavior began to emerge in literature at the height of the numerous corporate scandals at the beginning of the present millennia. Commonly known as the emerging leadership forms, this VBL comprising authentic, ethical, and servant leadership aims to address the question about providing value (i.e., ethical, moral, responsible, serving, and authenticity) in management (Lemoine et al., 2019). The definition of all three VBL forms has made explicit references to the manager’s impact on the wider organization. For example, managers who demonstrate servant leadership must acknowledge his/her moral responsibility toward the organization, follower, customer, and stakeholders (Ehrhart, 2004). In current times, this VBL is again gaining attention because businesses needs to continuously strive to serve and promote a positive outcome for their stakeholders. Despite the importance of these leadership, the concepts remain poorly understood from a behavioral lens and are often characterized in ways that only describe their importance for stakeholders (Hoch et al., 2018). A review on the literature also shows that two of these VBL (i.e., servant and authentic leadership) have multiple definitions. The widely shared concepts across all three leadership behaviors thus presented several critical issues. As such, this paper aims to answer the call of the special issue by discussing a new theoretical framework that influences the emergence of a VBL role, particularly, at the frontline of management.

First and foremost, VBL faced concept redundancy, given the existence of a plethora of commonalities that their definition shared. To illustrate, Table 1 highlights the different patterns of behavior that is embodied by the respective leadership theory. It shows that both authentic leadership and servant leadership have multiple definitions and that the prior conceptual definition of authentic leadership has continued to emphasize the role of the leader through their central attributes, for example, (a) the role of the leader as the central component to their self-concept, (b) achieved a high level of self-resolution or self-concept clarity, (c) their goals are self-concordant, and (d) their behavior is self-expressive (Shamir and Eilam, 2005, p. 399). In recent times, Eva et al. (2019) have attempted to redefine servant leadership by linking the leadership behavior to its outcome. As a result, the authors have emphasized on a greater self-sacrificing behavior and downplaying antecedents like personality to simplify the concept through its motive, mode, and mindset.

**TABLE 1:** The conceptual definitions of value-based leadership.

	Conceptual definitions
**Authentic leadership**	“A process that draws from both positive psychological capacities and a highly developed organizational context, which results in bot greater self-awareness and self-regulated positive behaviors on the part of leaders and associates, fostering positive self-development” (Luthans and Avolio, 2003, p. 243).
	“a pattern of leader behavior that draws upon and promotes both positive psychological capacities and a positive ethical climate, to foster greater self-awareness, an internalized moral perspective, balanced processing of information, and relational transparency on the part of leaders working with followers, fostering positive self-development. Note that this definition reflects several assumptions that underlie our perspective of authentic leadership” (Walumbwa et al., 2008).
**Ethical leadership**	“The demonstration of normatively appropriate conduct through personal actions and interpersonal relationships, and the promotion of such conduct to followers through a two-way communication, reinforcement, and decision-making” (Brown et al., 2005, p. 120).
**Servant leadership**	“The servant-leader is servant first… the differences manifests itself in the care taken by the servant – first to make sure that other people’s highest-priority needs are being served… do those served grow as persons? Do they, while being served, become healthier, wiser, freer, more autonomous, more likely themselves to become servants? And what is the effect on the least privileged in society; will they benefit or, at least, not be further deprived?” (Greenleaf, 1977, pp. 13-14).
	“Servant leadership is an (1) other-oriented approach to leadership (2) manifested through one-on-one prioritizing of follower individual needs and interests, (3) and outward reorienting of their concern for self toward concern for others within the organization and the larger community” (Eva et al., 2019).

Ethical leadership, on the other hand, was strongly influenced by the research of several scholars (see Treviño et al., 1998; Treviño et al., 2000, 2003; Den Hartog, 2015). This gave ethical leadership a stronger synthesis in its normative appropriateness definition, which focuses on the manager’s conduct when promoting its benefit to stakeholders. Meta-analytic paper has also show that ethical leadership is link to follower’s normative conduct even after accounting for job satisfaction (Peng and Kim, 2020). Therefore, ethical leadership was shown to benefits an organization by increasing and decreasing follower’s normative and counter normative conduct accordingly. However, ethical leadership normative stance has invited question about what norms the leader might refer to when choosing to promote them to followers. For example, favoring profit generation at the expenses of sustainability and fairness “would mean breaking the norm, rather upholding it” (Eisenbeiss, 2012, p. 793).

Furthermore, a recent work has argued that ethical leadership behavior is prone to retrospective bias (Banks et al., 2020, in press). However, we argue that such an issue is not limited to ethical leadership. Instead, all three VBL behaviors will face similar an issue of being simplified through follower’s evaluation. According to Epitropaki et al. (2013), followers will rely on cognitive simplification to cope with complex information processing. This is especially prevalent for these VBL because it is well associated with a top-down processing that often requires followers to interpret the presented value of their leaders, given that VBL circles around the concept of morality to address questions like sustainability, responsibilities, and justices. The ability of followers to interpret these shared believe about leadership behavior is critical to embedding the respective value in a social organization. To illustrate, work has shown that ethical leadership influence comes from the top and affect multiple levels of a formal organizational system through a cascading effect (Kuenzi et al., 2020). However, a top-down process faces constrains from factors such as the knowledge of past and concurrent behaviors to serve as an interpretation of the respective value behavior (Lord et al., 2020). For these reasons, scholars have called for research to define a set of normative reference point and conceive the meaning through an organization-wide phenomenon if value is indeed embedded in a social organization (Kahn, 1990; Klein, 2002).

Second, a review of existing literature shows that managers with a strong moral devotion will tend to do better in promoting positive organizational behavior through their moral image (see Jennings et al., 2015). Yet most research to date has mainly focused on the importance of VBL through their consequences rather than explaining the manager’s motivation for demonstrating and promoting these behaviors. Although all managers must demonstrate and promote moral values to entice their follower’s ethical behavior (Weaver et al., 2005), the overarching focus on positive consequences does not always explain why they will always emerge in a complex organization. As a result, consequential research has not adequately explained the conflation between the manager’s behaviors and their values, traits, and behaviors (Alvesson and Einola, 2019). Given that leadership is a two-way process that requires followers to appraise the leadership behavior to legitimize their influence, as well as if their behavior met the objective of the organization. Lord et al. (2017) argued that a collective identity (see DeRue and Ashford, 2010), in some part, must be made available within and between all levels of an organizational leadership system. Thus, how managers coordinate their thoughts and actions repertoire to meet the demand of an organization to demonstrate and promote VBL continues to highlight a limitation in literature.

Third, the emergence of a VBL behavior swells into the wider discourse and challenges faced by an organization. For this reason, a higher-level VBL behavior has been shown to trickle-down the organization (Mayer et al., 2009; Schaubroeck et al., 2012; Hirst et al., 2016; Wang et al., 2018a, b; Stollberger et al., 2019). The trickle-down model argues that higher-level management leadership (the source) is transferred to the lower-level management (the recipient) through the middle-level managers (the transmitter). In other words, “the perceptions, attitudes or behaviors of one individual can influence the perceptions, attitudes, or behavior of a second individual, which then influence the perceptions, attitudes, or behavior of a third individual” (Wo et al., 2015, p. 1848). Accordingly, the model aims to argue the role of higher-level leadership from one individual to another (i.e., A → B → C) and has primarily focused on the indirect influence (Bass et al., 1987).

The trickle-down model shows that management at different levels must display similar attributes for value to be transferred across an organization (De Cremer et al., 2018; Wo et al., 2019). Therefore, most research has leaned heavily on social learning theory (Bandura, 1977) to explain this cascading phenomenon. It is suggested that a role-modeling process will occur across different management levels, whereby “followers” (which refer to any employee that answers to a higher authority) will role model after their superior, in turn, allowing the value to “flow (or cascade) down” the organization (Mayer et al., 2009). Yet recent organizational scandals, such as the Kobe Steel (see Aizawa, 2018) and British Petroleum (see Amernic and Craig, 2017) and among many others have revealed a fragmented connection between top management’s (i.e., chief executive office, top-management team, and board leadership) ethos and lower line management behaviors. Especially, in very large and complex organizations, the image of the top management is often outwardly portrayed (see Peloza et al., 2012) to set the branding image of the organization for stakeholder. Thus, the reputation of organizations through its top management may fail to portray the reality of its internal organizational behavior, in particular, at the lowest level of management.

We have highlighted these issues to draw attention to the poorly understood emerging nature of VBL, in particular through this trickle-down model, although VBL would emerge in a social organization when the values are well communicated and shared. The influence of the wider organizational context in spiraling its emergence, as well as its effectiveness, remains limited (Lord and Maher, 1990; Liden and Antonakis, 2009; Day, 2012). For example, followers’ felt responsibilities under an ethical leader is found to have weakened when followers’ shared perception of moral awareness is high (Kalshoven et al., 2013). Such findings then question the merit of VBL behaviors as the sole contributor to positive organization behavior if the shared perception of ownership toward the organization can hinder its effectiveness. Furthermore, a manager’s behavior must also match the agentic prototype that is subscribed by the organization (see Gerpott et al., 2019), before they can shape the discourse of the wider organizational behavior. Based on such observations, we believe a comprehensive literature analysis utilizing that the role theory will help to address the aforementioned questions and to clarify the conditions that would support their emergence.

Accordingly, our paper aims to advance knowledge about VBL behavior emergence through three questions: First, why are VBL behaviors relevant at the frontline of management? Second, how are VBL behaviors sustained in a complex organization? Third, what is the framework that supports the development of frontline management VBL behavior? We draw on the role theory (Kahn et al., 1964) to underpin our analysis and argue that role is held in an organization that influences an individual’s attitude and behavior through the process of socializing (Sluss et al., 2011). The general perspective of role theory provides a foundation for role-related behaviors; for example, the role is defined as a set of interdependent behavior expectations (Katz and Kahn, 1978). As such, individuals who answer to the prescribed role of an organization will set up their own identity, which then influences their self-concept and their working relationship (Sluss and Ashforth, 2008). This suggested that an individual will develop the behavior expectation that is associated with the position that (s)he occupies (Burke, 1991).

A role theory perspective further extends social learning theory that has, in the past, been used to explain the interactional relationship that transpires the emergence of VBL behaviors through the trickle-down model. The social learning perspective suggests that leadership behavior is learned by role modeling after their direct managers demonstrate and promote VBL behaviors (Bass et al., 1987; Mayer et al., 2009). However, organizations often have informal groups, which are guided by different values or norms that are formally implemented in the organization (Schein, 2010). Lower-level employee may hence perceive and respond to these values differently due to their proximity from top management. Therefore, lower-level management plays an important role at instilling these policies, as well as influencing the moral emphasis of lower-level employee’s behaviors and decisions. Accordingly, Ruiz et al. (2011) suggested that formal authority of an individual will affect his or her attitude and behavior, making them aware of their role requirements that are set forward by top management. For this reason, management at different hierarchical levels understands their own role requirement (Bass et al., 1987) and plays a role in instilling the value from above and shape bottom-line perspective. Our current focused review thus contributes to theory in two ways.

First, we underline why and how VBL behaviors will emerge, particularly at the frontline managers’ level in an organization (Day, 2000; Gagnon and Collinson, 2014). Meta-analyses of ethical and servant leadership often approach frontline managers’ VBL behaviors to explain their positive effect on bottom-line follower’s behaviors (see Ng and Feldman, 2015; Bedi et al., 2016; Lee et al., 2020). Trickle-down research also suggested and found evidence that higher and middle management values will flow down and instill the value at the frontline of management. Therefore, the frontline managers will demonstrate VBL behaviors, as it is likely to inspire positive organizational behavior of their followers (Peng and Kim, 2020). Second, we provide a new theoretical framework to highlight the formal and informal processes that would allow VBL behaviors to emerge through the interaction between leaders and those who report to them. The proposed framework thus helps to understand how a frontline manager develops VBL behaviors. This allows future researchers to embrace the complexity of strategic management and the inherent role that managers have to perform in an organization (Georgakakis et al., 2019). Thus, as Antonakis (2017) stated, “finding different ways to study leadership is what will take our knowledge base to the next level” (p. 16).

In highlighting the aforementioned perspective, this paper will first explain the trickle-down model through the role theory perspective. We will then draw upon recent works of VBL to underline the processes, as well as the boundary conditions that are found to have strengthened VBL behaviors. We will conclude our review by highlighting future directions in the form of research propositions.

## Literature Search and Inclusion of Relevant Studies Over the Last 20 Years

We conducted a thorough literature search to identify published research that has examined the trickle-down model of VBL behaviors, as well as research that has examined VBL behaviors by drawing a role theory perspective. We searched for research that has been published in English between 2000 and 2020. We focused our review over the last 20 years because preliminary search across three databases only yields four papers between 1970 and 1999 that discussed about servant leadership (3) and authentic leadership (1). The papers are also much more abstract in nature rather than empirically testing the construct to provide evidence about the importance of VBL behaviors. In addition to servant and authentic leadership, the first white paper about ethical leadership only emerges in 2000, where it discussed the concept of a moral person and moral manager, that set the foundation pillars for ethical leadership (see Treviño et al., 2000). Nonetheless, we acknowledge that the concept of a trickle-down model of leadership has emerged in literature as early as 1978.

To ensure completeness, we used three electronic databases, which are EBSCOHost-Business Source Complete, Web of Science, and Scopus. We included the search terms “servant leader^∗^, ethic^∗^ leader^∗^, authentic leader^∗^, trickle^∗^, trickle-down, cascade^∗^” for publications related to the trickle-down framework in the title, keywords, and abstract. A total of 41 studies were returned, including journal articles, dissertations, and books. We excluded dissertations and books, as well as review journals to only focus on journal articles that have empirically examined the trickle-down model. As such, 29 returns were removed from our final selection and resulted in a total of 12 papers.

As for research on VBL behaviors and role theory, there are several known roles related or specific variants, such as role ambiguity, role clarity, follower role, role identity, and role identification, that researchers have used to categorize role theory (Zhao and Li, 2019). For the sake of parsimony, we excluded organizational identification and moral identity to enumerate characteristics that shape the role development of frontline managers’ VBL behaviors. The latter, moral identity has received widespread attention in many VBL behavior publications (see Aquino and Reed, 2002), underlining its importance as a moral antecedent that supports the development of VBL behaviors (see Jennings et al., 2015). We also excluded organizational identification to focus on role identities. Although Sluss and Ashforth (2008) argued that organizational identification can prime others to elicit similar responses, research has found it to inform unethical pro-organizational behavior at the expense of ethical leadership in financial institutions (Kalshoven et al., 2016). Furthermore, organizational identification has been well underscored by past research (see Mostafa, 2018) as an antecedent and/or consequences of role performance (see Riketta, 2005). Thus, we only focused on research that helps to strengthen the role expected behaviors to explain how the increasingly visible VBL behaviors are more likely to emerge in organizations through a role perspective.

We applied the search term “role theory, role perspective^∗^, role identity theory, servant leader^∗^, ethic^∗^ leader^∗^, authentic leader^∗^.” Our initial return yields a total of 380 research studies across all three electronic databases (i.e., EBSCOHost, Scopus, and Web of Science). In reviewing the research, many studies draw on social role theory and role congruity theory, which mainly focused on gendered leadership behavior. Because our current review aims to draw attention about the development of VBL behaviors through a role theory perspective, we decided to remove these research studies. Similarly, we filtered out dissertations and books to focus on journal articles that have empirically examined VBL behaviors through a role theory perspective or tested role mechanism (i.e., role ambiguity). As such, our final results were only 15 papers. Overall, this review frames our argument through the intersection of 27 research studies to pay attention to role theory and its implication on the trickle-down model of VBL.

## Role Theory

Since its inception, role theory has been used to highlight the phenomenon in complex organizations. Accordingly, role theory has been used to describe the role-making process that unfolds in dyads (Graen, 1976). Leaders are expected to communicate expectations, while their next level of staff will respond via an enhanced mutualexchange, trust, respect, and obligation (Matta et al., 2015). A role becomes more stable and routine when it is well communicated to develop shared perceptions (Graen and Scandura, 1987). Therefore, members of an organization must interpret their role expectations because a disagreement of own role expectation with those put forward by higher-level managers can result in competing role identities. As a result, members will enact different role behaviors (Farmer and Aguinis, 2005), going against the resources that were initially provided by the leader.

This differing role expectation can accentuate as a result of further social interaction, given that organizational members are often required to assume a set of patterned behaviors when they join the organizations (Biddle, 1979). Role theory argues that members who inhabit these social roles in an organization will align themselves with the expected rules and norms. The notion of role-taking behaviors hence suggests that organizational members, in particular, frontline managers, will attempt to maintain order due to the defining characteristic of the organizations (Mead, 1934; Katz and Kahn, 1978). This shows that role is often closely linked to the expectations set forward by higher management and will influence the views and behaviors of the role occupants. Nonetheless, the social expectations of the role will carry a moral value expectation, and this is widely accepted that job should be characterized with some ethical components (Downie, 1968). Thus, the role value cannot be divorced entirely from the role expectation as well as the behaviors of the role holder.

Role theory also differs from social role theory (Eagly, 1987; Eagly et al., 2000), which tends to classify the role played by the leader and the situation that clusters around gender and politics to accentuate social exchange obligation (Kacmar et al., 2011). In contrast to social role theory, frontline managers who participate in a social structure (i.e., joining the organization as an employee) must develop shared expectations, or they might face a conflict in their prescribed role expectation. Accordingly, a functional approach toward role theory suggests that “role” is conceived through shared normative expectations to explain behaviors within a social structure and system (Biddle, 1986). For this reason, Mead (1934) argued that roles will evolve through social interaction and allow the role occupier to interpret their own and other’s conducts through informal interaction. This in turn fosters role conformity through increase associated with the organization’s value and belief.

We argue that VBL behaviors will emerge through an interpersonal relationship that provides them with the opportunity to focus on developing skill and motivation, as well as targeting the welfare of the collective. Although leadership role is espoused through being in a formal and legitimate position, frontline managers’ interaction across the network of relationship can influence their self-concept and the way they behave (Katz and Kahn, 1978). Their knowledge of this self-concept can be elaborated through an informal and interpersonal relationship that serves as a strong indicator about their expected role. For example, research has found that employees enhance their conscientious personality when transitioning toward a managerial role to manifest the job demands of their new role (Li et al., 2020). This suggests that organizations have an equal role to play by ensuring that an employee’s contractual obligation is upholding to the highest standard when transitioning to a managerial role. For this reason, frontline managers become much more satisfied with their new role and much more willing to develop the role expected behavior. This is also known as a role choice behavior that is affected by the structural factor, such as legitimate position and status (Sluss et al., 2011). The informal relationship that develops at work could thus explain why frontline managers are willing to undertake extra-role responsibility such as challenging the organizational processes (see Venkataramani et al., 2016).

## A Role Perspective on the Trickle-Down Process of Value-Based Leadership Behavior

Attention on VBL behaviors has continued to grow due to increased interest in positive leadership that facilitates moral behaviors in organizations. Although there has been a rise in interest, many scholars have adopted an individual (or micro) perspective when arguing about the importance of the VBL behaviors (Lemoine et al., 2019). Hoch et al. (2018) stated that these emerging VBL behaviors often focus on the interpersonal dynamics that increase follower’s positive prosocial behaviors. Central to this approach is then directed through role modeling after higher-level VBL behaviors to promote socially acceptable and extra-role behaviors (Avolio and Gardner, 2005; Brown et al., 2005; Hu and Liden, 2013). However, this does not always explain the organizational condition, as well as why VBL behaviors will emerge in an organization (Solinger et al., 2020 in press). In stating the aforementioned perspective, we thus pay attention to the emergence of frontline managers’ VBL behaviors because these managers tend to be the focal point when augmenting moral behaviors (Peng and Kim, 2020), through the trickle-down model (Wo et al., 2019).

Table 2 provides a summary of the research that has examined the trickle-down effect of VBL behaviors. We apply role theory on the trickle-down effect because it extends the social learning model and strengthens our understanding about frontline managers’ role. The cascading process is also important in the field of VBL behaviors because frontline managers are not always well aware of the expectation that goes beyond their formal role responsibilities in the organization. For example, the social structure where these managers are organized can affect their perception of VBL behaviors, in particular, if there is any inconsistency in their legitimate status and role (Stryker and Serpe, 1982). Therefore, organizations often emphasize the importance of frontline managers when strengthening role value behaviors in an organization. Thus, Peng and Kim (2020) stated that frontline managers tend to have fewer resources due to the lower quality of social relationship with their higher-level managers (p. 361).

**TABLE 2:** Cascading research and its outcome.

Authors	Theory	Mediator	Condition	Core findings
**(8) Authentic leadership**				
Hirst et al. (2016)	Social learning/social exchange/relational helping behavior			Team leaders’ authentic leadership mediates the relationship between departmental authentic leadership and individual-level leader–member exchange (LMX). The result also shows that intra-team trust completely mediates the influence of team authentic leadership on both team helping behaviors and individual-level supervisor-directed helping behavior. The results reveal that self-concordance mediates the influence of team authentic leadership on individual-level supervisor helping behaviors as well as the influence of individual-level LMX on individual-level supervisor-directed helping behavior.
**(8) Ethical leadership**				
Mayer et al. (2009)	Social learning/social exchange			The results show a direct negative relationship between both top management and supervisory ethical leadership and group-level deviance, and a positive relationship with group-level organizational citizenship behavior (OCB). The effects of top management ethical leadership will trickle-down on group-level deviance and OCB, mediated by supervisory ethical leadership.
Schaubroeck et al. (2012)	Social learning	Unit ethical culture		Ethical leaders embed shared understandings through their influence on the unit ethical culture at various levels and, in turn, influence followers’ ethical cognitions and behavior. Ethical leadership will occur directly among immediate followers within a unit and indirectly across hierarchical levels through the cascading of ethical culture and senior leaders’ influences on follower leader behavior.
Hansen et al. (2013)	Social exchange	Employee relationship with organization; LMX		Different types of social exchange relationships would mediate these relationships; the within-foci effects (e.g., the relationship between organizational ethical leadership and commitment to the organization) are stronger than cross-foci effects (e.g., the relationship between supervisory ethical leadership and commitment to the organization). In contrast to the “trickle-down” model of ethical leadership, the results suggested that organizational ethical leadership is both directly and indirectly related to employee outcomes.
Ruiz et al. (2011)	Social exchange/role-set/resource-based			Top manager ethics will partially trickle-down to influence follower positive job response (job satisfaction, organizational commitment, turnover intention, and organizational citizenship) via the immediate supervisor. However, the effect of immediate supervisor is stronger for job satisfaction.
Byun et al. (2018)	Social exchange/social learning		Lower-level leader’s self-enhancement motives	High-level ethical leaders will trickle-down and reduce employee social loafing while increasing their task performance via lower-level ethical leader. Self-enhancement motives of low-level leaders were also found to moderate the relationship, strengthening this relationship when the motives are low rather than high.
Wang et al. (2018b)	Social learning/social exchange	Supervisor’s ethical efficacy expectation; supervisor’s ethical outcome expectation		Middle-level supervisor’s ethical efficacy expectation and unethical behavior–punishment expectation accounted for the trickle-down effect, while middle-level supervisors’ ethical behavior–reward expectation was not supported.
O’Keefe et al. (2019)	Social learning		Organizational ethical climate, organizational justice	Negative perceptions of organizational climate and justice increased the trickle-down effect of ethical leadership. The counterintuitive finding may be due to differences in situational strength between higher- and lower-level leaders; for example, less consensus at lower levels leads to unclear norms around ethics and justice and greater reliance of leadership for guidance.
Mozumder (2018)	Social learning/social exchange	Management trust (top management; middle management; supervisor)		The results show both downward and upward roles, where trust in leaders and ethical leadership were found to cascade across hierarchical levels and affect employee well-being and satisfaction. The results further showed that such positive effect can contribute to group OCB and organizational performance.
**(8) Servant leadership**				
Ling et al. (2016)	Service profit chain theory			Top-level servant leadership will trickle-down and enhance frontline employee service-oriented behaviors and service quality via middle-level servant leadership. This relationship is also moderate by the group service climate, strengthening the influence of middle-level servant leadership.
Wang et al. (2018a)	Social learning		Manager and supervisor organizational embodiment	Manager servant leadership will promote employees in-role and extra-role service performance via supervisor’s servant leadership. The relationship between (a) manager and supervisor servant leadership and (b) supervisor servant leadership and employee in-role and extra-role service performance is strengthened when their respective organizational embodiment is high.
Stollberger et al. (2019)	Role motivational/prosociality at work		Supervisor family motivation	The results show that manager servant leadership will trickle down and inspire supervisor servant leadership, in turn increasing employee prosocial motivation and subsequent work performance. However, supervisor family motivation buffers the trickle-down mechanism, such that the effect on employee work performance is weaker for supervisors with high levels of family motivation.

We further argue that the frontline managers will reflect on the norms, attitudes, and contextual demands to carry out the definition of their prescribed role. However, their perception can sometimes be misaligned due to idiosyncratic interpretations (Merton, 1957; Katz and Kahn, 1978; Zohar and Polachek, 2014). For example, research on idiosyncratic deals (or I-deals) is often context-specific through a voluntary agreement and non-negotiated nature of both parties (Rousseau, 2005; Rosen et al., 2013). We highlight this particular attribute because research has shown that higher-level manager’s servant leadership attributes will enhance this development and shape frontline managers’ perception by disentangling information from the wider organization (Rofcanin et al., 2018). It shows that a well-communicated role expectation from higher-level management is thus capable of strengthening frontline managers’ VBL behaviors because they see themselves as being a member of the organization (Ashforth, 2001).

Indeed, Bordia et al. (2010) stated that the trickle-down effect will uncover the role of higher-level managers as an antecedent to pattern similar behaviors to another manager who responds to their VBL behaviors. We extend this perspective by suggesting that role theory helps to underline a social structure in an organization to inform the behavioral expectation (Mead, 1934). For example, frontline managers’ repeated interaction with the environment helps to define their attitude and behaviors through vis-à-vis social interaction with other occupants of similar roles (Biddle, 1986; Reay et al., 2006). In other words, having a well-defined role will help an organization to embed VBL behaviors and allow them to emerge as a result of responding to the higher-level VBL behaviors (Eisenbeiss and Giessner, 2012). To illustrate such perspective, authentic leadership is found to enact authentic fellowship by satisfying basic needs and improving work role performance (Leroy et al., 2015). This shows that frontline managers can and will rely on the informal relationship, guided by VBL behaviors at work to improve understanding of their leadership role.

According to research that has examined the trickle-down effect of VBL behaviors, higher-level management is the antecedent that set the value tone on top to attract next-level management to develop similar VBL behaviors. Ethical leadership has by far received the largest attention because the seminal ethical leadership theory has highlighted the higher-level management role when embedding the values in an organization (Treviño et al., 2000, 2003), as well as spurring the development of frontline managerial behaviors (Mayer et al., 2009). For example, ethical leadership at the top and, in turn, ethical leadership in the middle are suggested to shape frontline managers’ ethical leadership behavior (Schaubroeck et al., 2012). More importantly, this cascading down effect will deter misconduct at the frontline of an organization (Mayer et al., 2009). This shows that ethical leaders are more likely to emerge at the frontline when the role expectation of managers is well defined across every level of the organization (Kuenzi et al., 2020).

Servant leadership at the top also appears to shape frontline managers’ servant leadership behavior (Liden et al., 2014) and influences employees’ prosocial motivation (Stollberger et al., 2019), as well as in-role and extra-role service performance (Wang et al., 2018a). The premise of servant leadership suggests that such leader behaviors will inspire stewardship toward a community (Greenleaf, 1977, 2002), and their commitment toward establishing next-level empowerment and growth to show that serving attributes and behaviors can transpire across multiple levels to enable fulfillment and personal ambition (Liden et al., 2008). This shows that servant leaders at the top of an organization will inspire serving behaviors of frontline managers and allow them to focus on addressing follower’s needs (Lee et al., 2020). For these reasons, servant leadership trickle-down research has found servant leadership behavior to trickle-down and strongly affect frontline service behaviors and performance (Ling et al., 2016).

However, authentic leadership trickle-down has only so far shown that a departmental authentic leader can affect team authentic leadership, leading to an increase in leader–member exchange via an intra-team trust and self-concordance (Hirst et al., 2016). Nonetheless, self-concordance is the extent where an individual is willing to pursue a goal that is consistent with their value and beliefs (Sheldon and Elliot, 1999; Sheldon and Houser-Marko, 2001). More often, enacting personal values and beliefs are associated with the perception of own role responsibilities, believing in its importance (Shamir and Eilam, 2005). For this reason, it shows that having an authentic leader higher up the hierarchy would signal role expectation about transparency, giving the frontline managers a purpose at work (Hirst et al., 2016). This descriptive attribute would thus inform role expected behaviors through a shared understanding of the value depicted by higher-level management (Katz and Kahn, 1978).

We argued that role theory will compliment social learning theory and, in turn, will promote VBL behaviors at the forefront of management (Bass et al., 1987; Mayer et al., 2009), because informal groups exist in organization and will impact the value of these bottom-line employees (see Schein, 2010). Management at the lowest level may perceive these values lesser than in their counterpart at the higher level (Treviño et al., 2008). The formal authority of an individual is also capable of affecting their role requirement awareness set forward by top management. Therefore, although role theory is often used in leadership research, our current review draws upon a role theory perspective and present the following proposition:

**Proposition 1:** Higher-level manager’s VBL behaviors will affect frontline managers’ value-providing roles. This in turn is expected to increase frontline managers’ willingness to demonstrate and promote VBL behaviors in an organization.

Further, research that examines the cascading trickle-down effect of VBL behaviors has continued to adopt the social learning perspective, which limits our understanding about employee’s responsibility toward the organization (Oldham et al., 1976), while attention is given to research that has linked the salience and activation of the role occupant to provide an understanding of how to particularize this relationship (Sluss et al., 2011). The trickle-down effect approaches the notion of providing the frontline managers with an understanding of own role expectation through higher-level VBL behaviors. However, the assumption that frontline managers are aware of their role obligations through a static contract that lays out their responsibilities and the behaviors to conduct the role (Kerr, 1978) does not always consider how arrays of other non-work behaviors can change the perception of their role behaviors (Wickham and Parker, 2007). Frontline managers who experience unfairness about their contract may become less willing to develop VBL behaviors, and this perspective centers around how frontline managers view organizational support (see Taylor et al., 009). Thus, in the next section, we will first synthesize the research that has examined VBL behaviors through a role theory to draw attention to the mechanism and boundary conditions that will shape the frontline managers’ role in the organization. Lastly, we will provide discussion about psychological contract breach and the role of human resource (HR) practices as future research avenues.

## Value-Based Leadership Behaviors and Role Theory

According to Paterson and Huang (2019), ethical leaders that demonstrate ethical voice will enhance the understanding of the next-level ethical role requirements, incorporating behavioral repertoires in an organizational setting. As such, an ethical leader is seen as the primary resource in providing an ethical basis for the role expectation. Such a perspective is consistent with organizational theories that focus on understanding how members of an organization will socially construct reality at work (Klieman et al., 2000). This perspective is also shared by research that found that managers who communicated ethical guidelines to reduce non-normative behaviors shaped the organizational norms and standards about ethical conduct (Hassan et al., 2020). It is suggested that individual beliefs about how others expect them to behave in a particular role will have the strongest influence on their judgment and decision-making capacity. The fear of social disapproval will, therefore, drive the needs for frontline managers to develop VBL to fuel the expectation of the social norm (Hassan et al., 2014). This suggests that members are perhaps more likely to report behaviors that go against the norm when other members of their group also demonstrate similar patterned behaviors (Mayer et al., 2013).

However, research has found that group competition climate tends to strengthen the indirect influence of servant leadership on service performance via self-efficacy, but not identification (Chen et al., 2015). Through role theory, it is suggested that the presence of a competitive climate will interfere with the frontline managers’ identification through an increasing need to compete (Friedkin and Simpson, 1985). This, in turn, will make it difficult for them to balance their interaction with colleagues due to the need to perform better despite answering to higher-level servant leaders (Chen et al., 2015). This aforementioned role perspective is also absent in authentic leadership literature. As an authentic leader is often distinguished through being (in)authentic, the degree where the individual and role would merge provides a salience expectation of the leadership role (Shamir and Eilam, 2005). As a consequence, research has made leader centrism as the heart of organizational functioning debate (Alvesson and Sveningsson, 2013). Organizational membership could play a significant role in legitimizing VBL behaviors (see Steffens et al., 2016), furthering our understanding about the mechanism that allows frontline managers’ behaviors to emerge in an organization. For this reason, the psychological interdependence between a focal colleague and the role occupant warrants attention, as it may help explain why the frontline managers would develop VBL behaviors and how competition can hinder their role identification.

Similarly, authentic leadership literature has focused on the authenticity of the leader’s behaviors via self-monitoring behaviors (Gardner and Cogliser, 2008). In contrast, the servant and ethical leadership focus on serving and ethical role, respectively (see Hoch et al., 2018). Authentic leaders must first perceive an authentic self-image before they can commit to role values (Quick et al., 2007). However, Neubert et al. (2013) argued that both authentic and ethical leaders share a common feature. For example, authentic leaders will equally present themselves with high moral standards to influence the next level of leaders and their respective role responsibilities (May et al., 2003). Their immoral behaviors also do not mean that they are inauthentic, but rather the issue of the role values tends to vary across an individual or contextual situation (Resick et al., 2011). Although this perspective reinstated the synonymity between both authentic and ethical leadership, the absence of a contextual influence on this VBL behavior has illustrated a paradoxical relationship between their authenticity and the role value (Sidani and Rowe, 2018). Hence, more research is needed to identify the boundary conditions where authentic leader’s role would emerge.

Research on role clarity, on the other hand, has shown to improve helping behaviors and reduce deviant behaviors under ethical leadership (Newman et al., 2015). While research evidence explains such relationship through social exchange theory, recent research suggests that exchanging relationship has roots in role theory (Matta et al., 2015). It is hypothesized that the interplay between leader and follower will provide proximal motivation, leading to an increase in quality engagement. For this reason, role clarity will increase salient behaviors and the willingness to respond to role values such as the investment of personal, physical, cognitive, and emotional energy (Rich et al., 2010). Indeed, when the frontline managers lack clarity about their role, it can affect their willingness to dedicate resources to a particular outcome like developing VBL behaviors. The limited resource also makes it difficult for the frontline managers to understand discretionary behavior, as well as their role responsibilities (Newman et al., 2015). Hence, the context as an impinging force can cause the frontline managers to deviate from the expected role and engage in behaviors that will not benefit the organization (Johns, 2006).

Accordingly, authentic leadership has been found to prevent role ambiguity and role conflict through an increase in affective commitment. It is suggested that when higher-level managers are transparent and trustworthy, categorized through their authentic nature, members are less likely to develop ambiguity and conflict in their role (Kalay et al., 2018). Likewise, servant leadership is shown to enhance both role and process clarity that increase team potency beliefs and enhance team performance and organizational citizenship (Hu and Liden, 2011). This implies that both types of leaderships will promote quality interaction, allowing shared beliefs about efficacy to emerge in a group to achieve general effectiveness (Guzzo et al., 1993). However, the findings of such research are not without limitations. For example, commitment and motivation toward developing expected role behaviors can increase the stress that discourages VBL behaviors. Thus, further attention is needed to better explain how role ambiguity or role clarity can increase (or decrease) strain that leads to reduced performance in a complex organization (Rizzo et al., 1970; Diebig et al., 2016). Based on the above analysis, we propose that:

**Proposition 2:** Role ambiguity and role conflict will affect the emergence of frontline managers’ VBL behaviors.

Overall, research that applied role theory to VBL behaviors has found leaders to influence moral concordance and compliance with the normative standards (Lemoine et al., 2019), while all three VBL behaviors tend to be categorized as homogenous to their approach (Dinh et al., 2014). The absence of boundary conditions further underscores the importance of understanding how both organizational and individual conditions can interact to inform role and influence VBL behaviors. Therefore, reviewing VBL behavior distinction and the boundary conditions will help underline the different foci to understand how the frontline managers will develop role expectancy behavior.

## Boundary Conditions that Strengthen the Role of Value-Based Leadership Behavior

Boundary conditions are paramount to our knowledge about why certain individuals are more likely to develop a stronger understanding of their role expectations. Accordingly, authentic followership is found to satisfy this basic need, translating to an increase in work role performance under the condition of authentic leadership (Leroy et al., 2015). Value-based followership is presumed to emerge as a result of the interaction between the leader and follower (Avolio and Gardner, 2005). Specifically, the influence of a VBL behavior aims to develop next-level authenticity, promoting the same component that is present in their higher-level leader behaviors (Gardner et al., 2005). This then increases self-awareness, internal regulatory process, and relational transparency (Deci and Ryan, 2000), hence becoming an active recipient of a higher VBL behavior influence (Shamir, 2007).

The pessimistic nature of leadership behavior is also observed in research on ethical leadership and role. Although ethical leadership will improve role clarity, having a passive nature will decrease its influence on role clarity (Vullinghs et al., 2018). It shows that the recipient of VBL behaviors requires active participation. Besides, the nature of VBL behaviors would entail concern and responsibility for those who they lead (Lemoine et al., 2019). Being passive is then a contrasting effect in an emerging own role as a future leader. Servant leadership research has echoed the argument about an individual’s passive nature. For example, a high level of employee avoidance-oriented motivation is shown to reduce their felt responsibility for constructive changes, making them more likely to demonstrate prohibitive voice (Arain et al., 2019). This in turn makes them less motivated to develop role responsibility like voicing for the sake of the organization.

Choosing to voice to challenge the *status quo* is an extra-role behavior that is distinct from other forms of citizenship behaviors (Morrison, 2014). Particularly, this behavior is associated with having risk when attempting to challenge the existing organizational norms, the behaviors of colleagues, and other associative attitudes and behaviors. For this reason, leaving those who enact such behaviors is open to criticism and accusation of disloyalty (Wei et al., 2015). Yet most VBL behavior literature often aims to justify their importance by directing the increase of positive (or decrease if negative) organizational behavior. Indeed, not much research has considered passivism to explain why some frontline managers might fail to develop value behaviors despite the existence of higher-level VBL behaviors. This has limited the perspective in current scholarship (Eisenbeiss and Brodbeck, 2014), whereby more research is needed to understand how the passivist nature of frontline managers can deter their development of patterned VBL behaviors.

The co-producing influence through a two-way process has also largely been absent in the existing research (Brown et al., 2005), often depicting next level as a passive recipient of VBL behaviors (Oc and Bashshur, 2013). Although management status will challenge VBL behaviors, at the same time, it will inform frontline managers’ role. Frontline managers’ status is an important boundary condition; in particular, frontline managers who experience status threat are more likely to augment their behaviors to increase their influence (Zhang et al., 2020). For example, frontline managers who perceive a stronger status in the organization may be more willing to speak up without the fear of retaliation (Paterson and Huang, 2019). We need to examine this boundary condition and its influence on role expectancy behaviors because emerging research is starting to reshape how we approach the framing process of VBL behaviors (see Derfler-Rozin et al., 2016; Desai and Kouchaki, 2017; Yam et al., 2019; Chen et al., 2020). Thus, frontline managers’ willingness to voice and give voice is important to maintain their status and influence (Bienefeld and Grote, 2014), because the existence of VBL behaviors is meant to foster an increase in similar patterned behaviors (Kakkar et al., 2016).

On the other hand, in discussing the active nature of VBL behaviors, we must pay attention to identification mechanism. In this regard, Sluss et al. (2011) argued that role identification will steer the development of role expected behavior. Role identification emerges asa result of the role occupant identity interacting with personal position and interpersonal relationship with those whom they share the same role (Sluss and Ashforth, 2007). Role identification differs from organizational identification in the way it facilitates role identity and role choice behaviors and is not bounded by competition (Chen et al., 2016). Because organizational identification is bounded by the responsibility and loyalty toward the organization, hence, having stronger organizational identification can result in pro-organizational motive to the degree of being unethical (Umphress et al., 2010; Thau et al., 2015). For this reason, the identification mechanism should gravitate toward the respective role value (i.e., social responsibilities, moral value, and serving value) that an organization intends to promote.

According to May et al. (2015), moral identification, which is the moral value depicted by the organization, can increase commitment and reduce turnover intention, especially in an organization that fulfills its legal compliance. It shows that employees who strongly associate with the moral value of an organization are more likely to carry themselves morally and strive to develop role expected behaviors by putting their thoughts and actions into practice. As a result, having a decree of moral identification would embed the expected role values through social stratification (Graham et al., 2011). This attribute is also important when explaining why the frontline managers would develop VBL behaviors when answering to higher-level VBL behaviors. However, no known paper to date has examined this perspective. Thus, research is needed to understand the employee’s value-based identification mechanism to explain how role expectancy behaviors can be promoted at the workplace (May et al., 2015).

The authentic leadership literature would narrate the influence of identification mechanism differently. It is suggested that an authentic leader will develop stronger relational identification based on their role identity and that this relationship is strengthened when their leader–member exchange is high (Niu et al., 2018). This further implies that relational identification is a precondition that shapes organizational identification rather than vice versa, which provides a salient view of the organization. The increase in the leader and member interaction would further bond their attribution, allowing them to develop role expectancy behaviors by answering to an authentic leader. More importantly, it underlines how the frontline managers’ role can be developed when they identify with higher-level manager’s value, making role conflict less likely (see Floyd and Lane, 2000).

To further our understanding of role identification influence, recent research examining environmental-specific servant leadership has provided an interesting understanding of green role identity. Green role identity is related to the concern about green-related resources, whereby exposure to environmental-specific servant leadership will fashion their environmental behaviors (Tuan, 2020). It is suggested that those who answer to this green VBL behaviors will find an alignment between their prosocial identity and the activity that reinforces their desire to display similar behaviors (see Gould-Williams et al., 2015). Their green role identity hence becomes a vital part of how they define themselves, and the nexus of both green role crafting and green role identity would be explained regarding how both cognitive and motivational resources would interact to inform role behaviors. Therefore, when the frontline managers perceive their role as befitting of their identity, they are more likely to advocate green communication (Tuan, 2020).

Last but not least, the role expectation of frontline managers can also shift according to the priority and benefits of the organization (Biddle, 1979). For example, the perception of moral ownership has a contagion effect that restrains creativity (Liu et al., 2020). Having such ownership is hence commonly associated with the desire to maintain the role value for the benefit of the organization (Treviño et al., 2014). Although ethical leaders will buffer role responsibility, they also reduce those with an inflated level of ownership from the burden of being monitored (Liu et al., 2020). This finding further highlighted the importance of role choice behaviors when explaining why VBL behaviors will emerge in an organization. Thus, Solinger et al., 2020 (in press) stated that delving into the role expectation will provide us with better clarity about how we can sustain the emergence of VBL behaviors in an organization.

The perspective of role theory is approached through several boundary conditions to depict personal resources. It mainly shows that passive behaviors can hinder the leader’s ability to pattern the leadership role from both authentic and ethical leadership (Leroy et al., 2015; Vullinghs et al., 2018). Likewise, having a high degree of avoidance motivation can diminish the effect of servant leadership on felt responsibility (Arain et al., 2019). Combining these different perspectives based on role theory allowed us to see how individual attributes can enhance the emergence of VBL behaviors. We also highlighted and argued that organizational identification is not always well associated with positive role expectation (Chen et al., 2016). This implies that frontline managers’ role and its myriad of (in)formal responsibilities must be well associated with the wider appraisal of the organizational context, as well as the interaction with own personal resource. In laying out the arguments, we would thus underline some key areas that future research could advance knowledge through this theoretical framework.

## Psychological Contract Breach as a Determinant of the Manager’s Role and Value-Based Leadership Behavior

In synthesizing the review through role theory, we underlined areas that need more attention, in particular, the capacity of the individual to interpret and display VBL behaviors (Liu et al., 2020). Emanating behaviors from the wider social environment require the frontline managers to associate their belief with the role value that is promoted/expected by the organization (Schepers and Van der Borgh, 2020). Negotiating and defining their role also require clarity and consensus. Therefore, when the role expectations are not congruent, the frontline managers may struggle to provide value while maintaining role expectations (Kahn et al., 1964; Katz and Kahn, 1978; Biddle, 1979; Quick, 1979). Soliciting the established role expectation is detrimental to the unwritten elements of the relationship between the frontline managers and the obligations of the organization (Rousseau, 1995), because role in organizations emerges through formal contractual negotiation that is of particular value to the role relationship (Katz and Kahn, 1978). Frontline managers will interpret this contract when establishing their understanding of the role expectation behaviors in accordance to the rules, norms, and procedures (Johnson et al., 2014; Lin and Johnson, 2015). Thus, when the set of agreements breach the role expectation, cognitive dissonance will emerge, resulting in adverse role behaviors.

Rusbult et al. (2005) argued that individuals, at some given point, will behave in a manner that goes against the role expectation by violating the norms that govern the role relationship. For example, the ethical leader has been shown to develop abusive tendency after crediting own moral behaviors from the previous day (Lin et al., 2016). The ability to balance the myriad of afforded resources to behave in accord to the role expectation in an organization is thus a reflection of line-managers’ ability to meet (or challenge) the role demands (Schepers and Van der Borgh, 2020). For example, an organization must fulfill its end of the contractual obligations to avoid discrepancies between the role holder and the role expectancy behaviors (Thomas et al., 2003). However, this interactional process often paints an incomplete picture by taking the perspective of the organization rather than the perspective of sole role occupant. This has limited our understanding of the motivation of the frontline managers to develop and provide VBL behaviors.

Indeed, organizations are often required to fulfill their end of the obligations by providing empowerment through their legitimate role. Psychological contract breach is a transactional relationship with varying levels of interpretation between the role holder and the organization (Thomas et al., 2003). The ability to behave in accordance to the role expectation can be defined through a set of agreements about the expectation of the organization and the role holder (Robinson and Wolfe Morrison, 2000). These expectations can also supersede role expectations such as promotion, training, and job security (Turnley and Feldman, 2000). Therefore, this dynamic relationship would govern how frontline managers execute their role and stresses the importance of psychological ownership as a result of their contractual role (Park et al., 2015; Liu et al., 2020).

Having psychological ownership toward the organization allows an individual to act as a value-providing agent (Hannah et al., 2011). Together with the support from the organization, Dutton et al. (2010) argued that such resources will strengthen their capacity at work by allowing them to deal with greater adversity and take advantage of newer opportunities. The ascription of role responsibility will further facilitate value-based decision-making (Treviño et al., 2014). For this reason, we theorize that the frontline managers would act beyond their agreed parameter (Rousseau, 1995; Morrison and Robinson, 1997) to provide extra-role behaviors, which allow VBL behaviors to emerge. Nonetheless, if frontline managers feel that their psychological contract has been breached, it will affect their role expectancy behaviors (Bordia et al., 2010). This may then translate to a decrease in wanting to display VBL behaviors. Thus, we propose the following proposition for future research.

**Proposition 3:** Psychological contract breach is negatively associated with lower frontline managers’ role value, which affects their ability to display VBL behaviors.

The expansive view of the psychological contract is also limited by our understanding of the boundary condition that could maintain the frontline managers’ role expectation. For example, we argued that role identity will inform frontline managers’ role choice behavior, as well as why they are more likely to be influenced by VBL behaviors (Zhu et al., 2016). However, critiques have argued that role value is often subjective to the social context (Resick et al., 2011). Accordingly, recent research has shown that a higher level of moral disengagement can shape the perception of the social context, sending problematic signals about the VBL behaviors and shaping next-level moral disengagement (Fehr et al., 2020). Although this process can be prevented by the existence of frontline managers’ moral identity, research has argued that VBL behaviors can either dampen the propensity of those with weaker moral identity or take on a corporative role for those with higher moral identity (Moore et al., 2019). For these reasons, research needs to pay attention to stable conditions like organizational resources to highlight the boundary conditions that allow VBL behaviors to emerge. Accordingly, we present the following research proposition.

**Proposition 4:** Frontline managers will display value leadership behavior when their moral engagement is strong. However, this relationship can be hindered when their perception of psychological contract breach is high.

## The Role of Organizational Human Resource Management Practices

Leader centrism has often been approached as the heart of an organizational functioning (Alvesson and Sveningsson, 2013). However, emerging research is starting to underline the role of the organization when supporting the development of frontline manager. It is suggested that adequate organizational support has been approached through many definitions, to name a few, supporting climate (Schepers et al., 2012), organizational participation (Rubel et al., 2018), and high-performing work system (Shen et al., 2014). However, the particular implication is placed on the HR management (HRM) practice, where scholars have argued that developing VBL behaviors should be a critical role for these practices (Blakeley and Higgs, 2014; Park et al., 2015).

We argue that these practices are pivotal to understanding the process and system that shape frontline managers’ VBL behaviors in the organization. However, so far, only a handful of research has examined its interactive influence. Therefore, scholars have often attempted to promote HRM practices as the condition that either accentuate or substitute the leader’s influence rather than provide an argument about its synergy (Kalshoven and Boon, 2012). Thus, we will take a broader approach in these areas to guide the development of the frontline managers’ role. Our call is partly synonymous with Leroy et al. (2018), suggesting that more research is needed to determine the nexus of HRM practices and VBL behaviors. However, our review differs by focusing on the emergence of VBL rather than the conditions that accentuate their influence, because those who struggle to be true to themselves as a result of the cultural and structural barriers are more likely to exit the organization when their role value fail to fit the expectation of the organization (Mayer et al., 2009; Gardiner, 2015; May et al., 2015). Hence, we stress on the importance of the organizational HRM practices and argue that policy and practices must provide a cohesive environment that affords frontline managers with the safety to express themselves in the workplace (Gardiner, 2017).

Yet within the discussion of leadership development, HRM practice tends to be confined to providing training (see Den Hartog, 2015), despite playing an important role in the fulfillment of the psychological contract (Kutaula et al., 2019) and managing psychological capital (Youssef and Luthans, 2012). The latter also underlines that attitudes such as hope, resilience, optimism, and efficacy are imperative for role expectation behaviors (Luthans et al., 2007; Peterson et al., 2011). Thus, it pays to lay the foundation where an organization can provide the space for the frontline managers to develop VBL behaviors. HRM is found to improve employee well-being and helping behaviors under ethical leadership when the perception of its practices is high (Kalshoven and Boon, 2012). Research on green HRM has also shown that such practice can interact with VBL behaviors and affect the organization’s environmental performance (Ren et al., 2020) and sustainability (Srivastava et al., 2020) and improve citizenship behaviors for the environment at both team and individual levels (Luu, 2019).

However, a major limitation in research is understanding how HRM practice could improve role alignment, specifically, how it can accentuate the frontline managers’ role expectation and their role choice behaviors through a systematic process (Leroy et al., 2018). For example, both ambidexterity and ethical leadership were highlighted as critical factors for knowledge sharing and team development competition (Liu et al., 2019). In building a service-oriented culture, the flexibility of HRM practices is also found to improve authentic leadership and job crafting behaviors (Luu, 2020). More importantly, the ethical leader is shown to complement HRM practices, improving affective commitment and reducing the intention to resist changes (Neves et al., 2018). As such, an organization’s HRM strategies concerning training and development can trigger a long-term trust, knowing that organizations will not diverge from their responsibility.

However, not all HRM practices will enforce ethical behaviors on equal footing. For example, research has found a negative interaction between high-commitment HRM and servant leadership on affective commitment and psychological empowerment (Stein and Min, 2019). A high-performing system that supports VBL behaviors is thus controversial (Boxall and Macky, 2009). This is in part because the extensive implementation of a high-performing system can increase work demand and is more likely to induce stress as a result of increasing work intensity (Godard, 2001). In line with our argument about the psychological contract breach, we argue that frontline managers must be afforded with the right condition to sustain their role expected behaviors (see Alfes et al., 2017).

Taking stock on this perspective, we argue that alignment between HRM practices and VBL behaviors can strengthen the relationship, reinforcing frontline managers’ willingness to reciprocate role expectation. HRM and VBL must develop a synergistic perspective to complement one another (Argyris, 1998). For example, research has shown that HRM systems that compromise ability, motivation, and opportunity (AMO)-enhancing practices can influence ethical work climate (Guerci et al., 2015). Accordingly, the AMO that portrays the HRM system as an additive index through three dimensions (Jiang et al., 2012) can help develop values-oriented programs to advance the organization value goals. These value-oriented programs tend to be successful when they help the organization to establish new values and ensure that members adhere to these values regularly (Weaver and Treviño, 2001). Therefore, the combinations of all three HR practices will thus affect the employees’ general satisfaction and underline the effectiveness of an organization (Katz et al., 1985).

The AMO model will also have a broader impact on the organization and influence the way members conduct their behaviors in a moral manner (Way and Johnson, 2005). Although the AMO model will not align the members toward specific value behaviors (Werbel and Balkin, 2010), it will affect the overarching value of the organization in which members are embedded (Guerci et al., 2015). In the context of VBL behaviors, the AMO model could offer us an understanding of how such practices can create the context for role expected behaviors to emerge. Individuals with the ability and motivation to carry out the role value are more likely to value the opportunity provided by the organization (see Weaver and Treviño, 2001). This relationship across three dimensions is also approached as a form of mutual exchange of investment on members who will benefit the organization’s frontline behaviors (Choi, 2014). Based on such assumptions, the following proposition is presented.

**Proposition 5:** Organizational HRM practices will diminish the frontline managers’ perception of psychological contract breach. This then mitigates the negative associations with lower frontline managers’ role value and their ability to display VBL behaviors.

## The Impact of Different Human Resource Management Practices on Value-Based Leadership Behavior

Each HRM practices can also impact value role behaviors differently (Guerci et al., 2015). The focus on work characteristics like work enrichment, autonomy, complexity, and control is important to tease out the potential HRM practices that would increase the willingness to participate through a two-way process role development process (Lee et al., 2019). Indeed, the HRM practice that provides the autonomy and opportunity must be closely associated with the human capital policy to underline its effectiveness of Den Hartog et al. (2013). HRM activities that are purely administrative are also unlikely to affect behaviors if they are not deemed effective (Choi, 2014). Therefore, the AMO model, specifically, opportunity-enhancing HR practices, can develop a unique relationship with frontline managers’ role expectation by encouraging them to develop VBL behaviors and can provide the opportunity for knowledge to flow across the organization (Chuang et al., 2016).

The intrinsic motivation for active involvement, flexible job design, teamwork, and information sharing (Jiang et al., 2012) will enthuse the frontline managers to enact their role expectation. Research has shown that opportunity-enhancing practices alone can help develop higher commitment (Gong et al., 2009), lower turnover intentions (Jensen et al., 2013), higher productivity and quality (MacDuffie, 1995), better service performance (Chaung and Liao, 2010), enhanced safety performance (Zacharatos et al., 2005), and better financial performance (Huselid, 1995). We argue that such an attribute is important for the frontline managers because these managers do not always have the opportunity to participate in the decision-making process. Often, frontline managers have to rely on their abilities to interact with environmental constraints and influence the expected organizational outcomes (López-Cotarelo, 2018).

The frontline managers also tend to avoid prescribing to a certain policy that contradicts the aim of higher policymakers to carve out a space of their own. This approach can then lead to a devolution of HRM practices due to centralized decision-making that emphasis procedural consistency due to association with day-to-day business management, because HRM practitioners do not always intervene due to the labor cost, especially in an industry segment where cost outweighs components of an organizational strategy (Boxall and Purcell, 2016). Therefore, different HRM practices might affect value role expected behaviors differently. For example, ability and opportunity-enhancing practices are found to influence benevolent and principle organizational ethical climates (Guerci et al., 2015). More importantly, both ability and opportunity practices were related to value and compliance-oriented programs (Weaver and Treviño, 2001). For this reason, the use of incentive rewards and rule expectation would signal the organization’s commitment to provides social norm and infrastructures to communicate value role behaviors.

However, research has also found that motivation-enhancing practices contrast the effort of both ability and opportunity-enhancing practices. Instead of developing conditions that enhance value behavior, motivational-enhancing practices are found to nurture self-interest (Guerci et al., 2015). Indeed, Weaver and Treviño (2001) argued that practices that develop based on punishment and reward cannot always guarantee to provide the condition for the emergence of value-providing behaviors. Individuals who are motivated by the rewards can develop self-interest behaviors rather than be motivated to conduct ethical behaviors. It is also important to note that some scholars have argued against such a proposition and highlighted the importance of motivational practices for organizational moral behaviors (see Winstanley and Woodall, 2006).

We argue that the opportunity to engage with other role occupants that share the same social space will provide clarities about their role expected behaviors (Sluss et al., 2011). This, in turn, creates a meaningful work experience that enhances their skills and motivation (Oppenauer and Van De Voorde, 2018) and the ability to cope with the role demands. The opportunity to share information can further develop complex learning behaviors that are important for the emergence of a specific phenomenon (Ployhart and Moliterno, 2011). For this reason, we propose that frontline managers’ perception of a psychological breach is more likely to decrease when an organization’s HRM practice provided opportunities (see Prieto and Pilar Pérez Santana, 2012; Patel et al., 2013; Park et al., 2019) that broaden their expertise to meet their end of the agreement. Hence, while it is important to approach HRM practices as one single entity through the AMO model, the particular contribution of each dimension (i.e., AMO) can impact value-oriented behaviors differently. Nevertheless, more research is needed to understand the role of HRM practices and how each dimension will affect the value-oriented behaviors of management (Boxall and Macky, 2009; Guerci et al., 2015). Hence, we propose that:

**Proposition 6a:** Ability-enhancing practices will diminish the frontline managers’ perception of psychological contract breach. This then mitigates the negatively associated lower frontline managers’ role value and their ability to display VBL behaviors.

**Proposition 6b:** Motivational-enhancing HRM practices will diminish the frontline managers’ perception of psychological contract breach. This then mitigates the negatively associated lower frontline managers’ role value and their ability to display VBL behaviors.

**Proposition 6c:** Opportunity-enhancing practices will diminish the frontline managers’ perception of psychological contract breach. This then mitigates the negatively associated lower frontline managers’ role value and their ability to display VBL behaviors.

In sum, we have paid attention to the AMO model; specifically, we underlined the implication for future research to better provide an insight into the relationship between HRM practices and the wider organizational behavior (Wood et al., 2012; Jensen et al., 2013). Although the majority of research tends to examine the AMO model as a whole rather than decomposing the system (see Vermeeren, 2017), providing knowledge about work autonomy and involvement in decision-making almost always pointed toward opportunity-enhancing HR practices (Boselie, 2010), because elements of an organization that accentuate role expectation and penalize it they failed to meet them (Lepak et al., 2006) will accentuate the frontline managers’ VBL behaviors. This inherited resource will also endow a higher-level VBL behavior as a strategic resource that is important for organizations that want to embed VBL behaviors (Wooldridge et al., 2008). Thus, we believe that the opportunity offered to frontline managers may very well support the emergence of the VBL behaviors and empowers them psychologically and diminish their perception of psychological contract breach.

## Discussion and Future Research on Value-Based Leadership Behavior and Role Theory

Our above literature analysis shows that most trickle-down research on VBL behaviors has mainly focused on understanding this effect through a social learning perspective (Wo et al., 2019). Although the frontline managers may not always have the opportunity to socialize with management up the hierarchy due to the distance between higher-level management (Antonakis and Atwater, 2002), numerous recent corporate scandals have highlighted a fragmented connection between upper management and frontline managers’ practices (see Amernic and Craig, 2017; Aizawa, 2018). Most literature continues to emphasize that VBL behaviors can be socially learned from managers up the hierarchy despite the existence of contingent role with the frontline managers and higher management being spread across distance and time (Yang et al., 2010). Thus, our review contributes to theory by highlighting how frontline managers’ VBL behaviors will emerge through a role perspective. In this regard, we have argued that the frontline managers’ VBL behaviors will emerge because of their role understanding in an organization. This in turn shapes their attitude and makes them more likely to display VBL behaviors.

We also contribute to the theory about the development of frontline VBL behaviors to explain their positive effect on follower’s behaviors (Day, 2000; Gagnon and Collinson, 2014).

As such, we argue that a frontline manager who demonstrates VBL behaviors will inspire next-level positive organizational behavior (Peng and Kim, 2020). In doing so, we offer a role theoretical framework to highlight the formal and informal processes that would allow VBL behaviors to emerge through the interaction between the leader and those who report to them. A role theory perspective is important because it helps us to understand how the frontline managers would define their role in an organization. It also offers practitioners an understanding of strategic management and the inherent role that frontline managers have to perform in an organization (Georgakakis et al., 2019).

The ongoing development at work has also significantly changed the relationship between employees and the organization. In acknowledging these implications, we argued that frontline managers’ perception of a psychological breach can affect their VBL behaviors, given that enacting VBL behaviors must be consistent and draws heavily on personal resources (Lin et al., 2016). In a situation when fairness is not reciprocated, it can impact frontline managers’ willingness to develop VBL behaviors and can hamper commitment and trust toward the higher-level managers and the organization (Alcover et al., 2017). Because psychological contract would play an important role and a crucial aspect in organizational life, we further argue about the role of HRM practices to provide an integration of the organizational support. Indeed, formal conditions as a result of policy and practice can facilitate the increase of role commitment when they are deemed fair by the frontline managers (Taylor et al., 2009). However, as not all HRM practices will impact the role equally, especially when it involves value-providing behaviors (Guerci et al., 2015), hence, we call for future research to disseminate the ability and opportunity and motivation-enhancing practices to determine each practice strength on frontline managers’ perception of psychological contract breach.

Nevertheless, from a practitioner perspective, it shows that organizations need to strategically allocate their resources to develop managers to inspire next-level (or future) leadership role at the frontline. VBL behaviors are often far more demanding than demonstrating managerial competency. To sustain the emergence of VBL behaviors across the organization, practitioners must develop a culture that promotes value role behaviors. Frontline managers are also more likely to be inspired to achieve value role behaviors when they feel that the organization properly communicates their role expectations. This review thus recommends that organizations should adopt training activities that develop VBL behaviors across multiple levels of management and, at the same time, develop policy and practices that enforce the understanding of these value norms. Organizations could also implement incentive and reward systems to ensure that frontline managers understand their role responsibility. For this reason, we contribute to knowledge by presenting future research directions where researchers can investigate the process that drive the emergence of frontline managers’ VBL behaviors.

## Conclusion

In closing, our current review calls for future research to examine the cascading model through a role theory perspective. In doing so, we discussed research that has examined VBL behaviors through role theory and synthesized the boundary conditions that could further our knowledge in understanding why frontline managers will develop VBL behaviors in an organization. We also presented directions for future research. Overall, we have argued that a role theory perspective warrants further investigation as VBL behaviors will not emerge in a vacuum. Thus, further attention must be paid to understand the organizational process and the boundary conditions that supported these leaders’ emergence.

## Author Contributions

All authors listed have made a substantial, direct and intellectual contribution to the work, and approved it for publication.

## Conflict of Interest

The authors declare that the research was conducted in the absence of any commercial or financial relationships that could be construed as a potential conflict of interest.
